# Male Sterility of an *AHAS*-Mutant Induced by Tribenuron-Methyl Solution Correlated With the Decrease of AHAS Activity in *Brassica napus* L.

**DOI:** 10.3389/fpls.2018.01014

**Published:** 2018-07-13

**Authors:** Jinyang Lv, Qianxin Huang, Yanyan Sun, Gaoping Qu, Yuan Guo, Xiaojuan Zhang, Huixian Zhao, Shengwu Hu

**Affiliations:** ^1^State Key Laboratory of Crop Stress Biology in Arid Areas, Yangling, China; ^2^College of Life Sciences, Northwest A&F University, Yangling, China; ^3^College of Agronomy, Northwest A&F University, Yangling, China

**Keywords:** rapeseed, male sterility, acetohydroxyacid synthase, *AHAS* mutant, hybrid seed production

## Abstract

Tribenuron-methyl (TBM), an acetohydroxyacid synthase (AHAS)-inhibiting herbicide, can be used as an efficient chemical hybridization agent to induce male sterility for practical utilization of heterosis in rapeseed (*Brassica napus* L.). Utilization of rapeseed mutants harboring herbicide-resistant AHAS alleles as the male parent can simplify the hybrid seed production protocol. Here we characterized a novel TBM-resistant mutant *K5* derived from an elite rapeseed variety, Zhongshuang No. 9 (*ZS9*), by ethyl methyl sulfonate mutagenesis. Comparative analysis of three *BnAHAS* genes (*BnAHAS1, BnAHAS2*, and *BnAHAS3*) between the mutant *K5* and *ZS9* identified a C-to-T transition at 544 from the translation start site in *BnAHAS1* in *K5* (This resistant allele is referred to as *BnAHAS1^544T^*), which resulted in a substitution of proline with serine at 182 in BnAHAS1. Both *ZS9* and *K5* plants could be induced complete male sterility under TBM treatment (with 0.10 and 20 mg⋅L^-1^ of TBM, respectively). The relationship between TBM-induced male sterility (*Y*) and the relative AHAS activity of inflorescences (*X*) could be described as a modified logistic function, *Y* = 100-*A*/(1+*Be*^(-^*^KX^*^)^) for the both genotypes, although the obtained constants *A, B*, and *K* were different in the functions of *ZS9* and *K5*. Transgenic *Arabidopsis* plants expressing *BnAHAS1^544T^* exhibited a higher TBM resistance of male reproductive organ than wild type, which confirmed that the Pro-182-Ser substitution in BnAHAS1 was responsible for higher TBM-resistance of male reproductive organs. Taken together, our findings provide a novel valuable rapeseed mutant for hybrid breeding by chemical hybridization agents and support the hypothesis that AHAS should be the target of the AHAS-inhibiting herbicide TBM when it is used as chemical hybridization agent in rapeseed.

## Introduction

Rapeseed (*Brassica napus* L.) is one of the most important oil crops worldwide, which not only provides edible oil for people but also supplies stable livestock meal, lubricants and biodiesel (Vollmann and Rajcan, 2010). Significant heterosis for seed yield and other agronomic traits has been well documented in rapeseed (Grant and Beversdorf, 1985; Lefort-Buson et al., 1987; Brandle and Pbe, 1990; Engqvist and Becker, 1991; Starmer et al., 1998; Qian et al., 2007, 2010). Several pollination control systems have been applied to produce hybrid seeds in rapeseed, such as cytoplasmic male sterility, genic male sterility, self incompatibility, chemical hybridization agents (CHA) induced male sterility (CIMS), and ecological genic male sterility (Fu, 1995; Fu et al., 2014). Compared with other approaches, the major advantage of CIMS is that almost any inbred line with desirable traits can be used as female parent in hybrid breeding program. Moreover, the male sterile phenotype cannot inherit, meaning that the heterosis can also be utilized for multiple generations. Hence CIMS has aroused great concerns in the utilization of heterosis (Guan, 2014).

Much attentions have been paid to exploit new chemicals that can induce male sterility in plants since Moore (1950) and Naylor (1950) reported their pioneering works that maleic hydrazide could induce maize male sterility. Some chemicals have been reported as CHAs for hybrid seeds production in rapeseed (Guan, 2014; Yu and He, 2014). A few of sulfonylurea herbicides, which are a class of the most widely used acetohydroxyacid synthase (AHAS, EC 2.2.1.6)-inhibiting herbicides, can induce complete male sterility in rapeseed at a concentration less than 1% of that required for their herbicide activity, such as tribenuron methyl (TBM) (Yu et al., 2006; Zhang et al., 2010; Yu et al., 2017), monosulfuron-ester sodium (Cheng et al., 2013, 2015; Li Z. et al., 2015), and amidosulfuron (Yu et al., 2009). So far, several dozens of commercial rapeseed (*B. napus*) hybrids based on male sterility induced by TBM or other sulfonylurea herbicides have been registered in China (Cheng et al., 2013; Yu et al., 2017).

AHAS, also known as acetolactate synthase (ALS, E.C.2.2.1.6), catalyzes the first step of the synthesis pathway of the branched-chain amino acids (BCAAs), including valine, leucine, and isoleucine (Umbarger, 1978; Duggleby et al., 2008). There are three functional genes, *BnAHAS1, BnAHAS2*, and *BnAHAS3*, encoding the catalytic subunit of AHAS in *B. napus* (Ouellet et al., 1992), which also are named as *BnaC.AHAS.a, BnaA.AHAS.b, BnaA.AHAS.a*, respectively (Li H. et al., 2015). Among the three genes, *BnAHAS1* (*BnaC.AHAS.a*) and *BnAHAS3* (*BnaA.AHAS.a*) are highly similar in sequence, and both are constitutively expressed genes that should have housekeeping functions. Whilst, *BnAHAS2* (*BnaA.AHAS.b*) is different from the other two, which is expressed only in mature ovules and the extra-embryonic tissues of immature seeds (Ouellet et al., 1992).

Five classes of AHAS-inhibiting herbicides have been widely applied to control weeds in modern agricultural production, including the sulfonylureas (SU), imidazolinones, triazolopyrimidines, pyrimidyl-thiobenzoates, and sulfonylaminocarbonyl-triazolinones (Ray, 1984; Shaner et al., 1984; Gerwick et al., 1990; Subramanian et al., 1990; Santel et al., 1999). These herbicides are confirmed to be potential inhibitors of AHAS by changing the active site into conformations that are hard to bind with substrates (Duggleby et al., 2008; Garcia et al., 2017). However, with the widespread application of these herbicides, plants (weeds) can evolve resistance phenotype commonly through AHAS mutation to survive and reproduce in the presence of herbicides (Powles and Yu, 2010; Busi et al., 2013). Based on *Arabidopsis thaliana* AHAS protein sequence, eight amino acid substitutions AHAS, including Ala122, Pro197, Ala205, Asp376, Arg377, Trp574, Ser653, and Gly654, have been reported in plant that confered resistance to one or more AHAS-inhibiting herbicides (Tranel et al., 2017). In *B. napus*, several AHAS-inhibiting herbicide-resistant mutants obtained by spontaneous mutagenesis, chemical mutagenesis using ethyl nitrosourea or ethyl methyl sulfonate (EMS), including PM1 and M9 that harbor Asp instead of Ser at position 653 of BnAHAS1 (named as Ser-653-Asp in BnAHAS1 for short), PM2 and M342 (Trp-574-Leu in BnAHAS3), and M45 (Pro-197-Ser/leu in BnAHAS3) (Swanson et al., 1989; Tonnemaker et al., 1992; Magha et al., 1993; Hattori et al., 1995; Hu et al., 2015, 2017; Li H. et al., 2015), have been reported to be resistance to one or more dissimilar AHAS-targeted herbicides. These *AHAS* mutants have been used to develop herbicide-resistant rapeseed varieties (Tan et al., 2005) or are recommended to be used as male parents in CIMS hybrid seed production (Li H. et al., 2015).

It has been confirmed that AHAS is the target of AHAS-inhibiting herbicides (Pang et al., 2003; Mccourt et al., 2005, 2006). Recently, it was found that a few SU-herbicides can induce complete male sterility in *B. napus* at a lower concentration (Yu et al., 2006, 2009, 2017; Cheng et al., 2013, 2015), this suggested that these SU-herbicides are potential CHAs in rapeseed hybrid breeding. Previous studies tried to explore the mechanism underlying CIMS (Cheng et al., 2013; Li Z. et al., 2015; Zhao et al., 2015; Liu et al., 2017). Zhao et al. (2015) indicated that foliar-sprayed TBM was polar-transported to anthers, resulted in the BCAAs starvation via anther-specific AHAS inhibition and autophagic cell death in anthers, and ultimately led to male sterility. This suggested that AHAS should be the target of CIMS.

In this study, we characterized a new TBM-resistant rapeseed mutant *K5* derived from an elite rapeseed variety *Zhongshuang No.9* (*ZS9*) through EMS mutagenesis. The objectives of the present investigation were: (1) to reveal molecular bases of TBM-resistance in the mutant *K5*; (2) to determine the relationship between the changes of AHAS activity and TBM-induced male sterility; (3) to reveal the target of TBM when used as a CHA.

## Materials and Methods

### Plant Materials and Chemical

Two rapeseed (*B. napus*) lines, *Zhongshuang No.9* (*ZS9*) and *K5*, were used in this investigation. *ZS9* was developed by the Oil Crops Research Institute of the Chinese Academy of Agricultural Sciences (Wuhan, China) and selfed for eight generations before being used in the present study. *K5* is a TBM-resistant mutant line derived from *ZS9* via EMS mutagenesis and obtained by TBM foliar-spray screening (Qu et al., 2014; Sun et al., 2015).

*Arabidopsis thaliana* (Col-0 ecotype) plants were grown at 22°C under a 16-h light/8-h dark cycle (light intensity 6000 ∼ 9000 lux) and approximately 60% relative humidity in phytotron.

Tribenuron methyl (TBM, MaiFa^®^, 10% active ingredients) used in the present experiment was produced by Hetian Chemical Co. Ltd. (Shanyang, China).

### TBM Treatment in Rapeseed Plants

*ZS9* and *K5* were planted in Yangling Regional Test Station of Crop Varieties, Shaanxi, China (N34.29°, E108.06°) at two growing seasons, in 2015–2016 and 2017–2018. No herbicides or pesticides were used before bolting stage. All the trials were managed in accordance with standard agricultural practices. Rapeseed plants at the bolting stage with the largest flower bud ≤ 2 mm in length and about at the uninucleate stage of pollen development (Yu et al., 2006) were first foliar-sprayed with different concentrations of TBM solution, and second foliar-sprayed 1 week after the first time.

For *K5* line, 11 concentrations of TBM solution (0, 0.05, 0.10, 1.00, 2.00, 5.00, 10.00, 15.00, 20.00, 30.00, and 40.00 mg⋅L^-1^) were used to foliar-spray, while for *ZS9* line, five concentrations of TBM solution (0, 0.05, 0.10, 1.00, and 2.00 mg⋅L^-1^) were used, and approximately 5 ml TBM-solution was used for per plant. The field trials were arranged in a completely random block design with three replications for each of the both lines. Each plot contained five rows, with 4.0 m in length, 0.5 m between rows, and 0.10 m between plants.

### Pollens Viability

The main inflorescences of five rapeseed plants treated with each concentration of TBM solution were separately collected and put into Carnoy’s Fluid at the beginning of flowering time. Alexander staining was carried out to determine pollen viability (Alexander, 1969), and micrographs were taken by a microscope (Olympus BX51, Japan). Sterility of pollens was estimated by calculating the number of sterile pollens in five plants from each treatment, using Image J 1.46r (Wayne Rasband, National Institutes of Health, United States). TBM-treated plants with more than five male sterile flowers were considered as male sterile in the experiment.

### Amplification and Sequence Analysis of *BnAHASs*

Genomic DNA of rapeseed *ZS9* and *K5* was extracted from 0.5 g young leaves by cetyltrimethyl ammonium bromide (CTAB) method and used as template for polymerase chain reaction (PCR) amplification. Primer pairs used for PCR amplification of *BnAHAS1, BnAHAS2* and *BnAHAS3* were designed as BnAHAS1-F (5′_CCCCCGGGTCTCCTCTAACCATGGCGG_3′) and BnAHAS1-R (5′_GGGTCACCAGCTTCATCTCTCAGTAC_3′); BnAHAS2-F (5′_CCCCCGGGAGCAATTTCTCGCAACACTC_3′) and BnAHAS2-R (5′_GGGTCACCAAATAAAGAGTGAAGTTTGCGT_3′); BnAHAS3-F (5′_CCCCCGGGCTCTCTCATCTAACCATGGC_3′) and BnAHAS3-R (5′_GGGTCACC GGTCGACGATTACTGAAACTA_3′), respectively, with restriction enzyme sites of XmaI and BstEII in the forward and reverse primers, respectively. The PCR reaction mixture of 50 μl contained approximately 50 ng genomic DNA, 1 × PCR buffer, 0.2 mM dNTPs, 1.5 mM MgSO_4_, 0.3 μM each primer, and 1U KOD-Plus-Neo (TOYOBO), and the PCR program was as follows: 2 min pre-denaturation at 94°C; 10 s denature at 98°C, 30 annealing at 58°C, and 1 min extension at 68°C for 40 cycles; and 10 min incubation at 68°C. The PCR amplification experiment was conducted three independent times for each of the three *BnAHAS* genes. The PCR products were ligated to pMD^TM^19-T vector (TaKaRa) after being added an ‘A’ nucleotide at their 3′-end, and then transformed into DH5α competent cells. Five positive clones from each PCR product of each *BnAHAS* were randomly selected for sequencing at Sangon Biotech, Shanghai, China.

The sequences obtained were analyzed using DNAMAN 6.0 (Lynnon Biosoft^1^). The *AHAS* sequence of *A. thaliana* (*At3g48560*) was downloaded from TAIR^2^. Multiple sequence alignments were performed using ClustalW 1.81 (Thompson et al., 1994).

### *Arabidopsis* Transformation and TBM-Treatment

To determine whether the allele *BnAHAS1^544T^* in *K5* was responsible for TBM- resistance or not, we constructed its expression vector CaMV35S:: *BnAHAS1^544T^* using pCAMBIA3301 as a backbone. We first modified pCAMBIA3301 by inserting an additional CaMV35S promoter into its multiple cloning site by digesting it with EcoRI and SacI (TaKaRa) and ligating with T4 DNA ligase (TaKaRa). Then, *BnAHAS1^544T^* from pMD19T-*BnAHAS1^544T^* was introduced into the modified pCAMBIA3301 by digesting with XmaI and BstEII (TaKaRa) and ligating with T4 DNA ligase. The constructed vector CaMV35S:: *BnAHAS1^544T^* was confirmed by sequencing, and then transformed into *A. thaliana* (Col-0) by *Agrobacterium tumefaciens*-mediated floral-dip method (Zhang et al., 2006). Positive transgenic plants were selected by foliar-spraying 0.1% Basta (20% glufosinate, Bayer) at the cotyledon stage. The finally obtained homozygous transgenic lines were foliar-sprayed with two concentrations of TBM solution (0.007 mg⋅L^-1^, 2.000 mg⋅L^-1^) at the bolting stage, approximately 2 ml solution per plant.

### RNA Extraction and Reverse Transcription-PCR (RT-PCR)

Total RNA of cauline leaves and inflorescences of the homozygous *BnAHAS1^544T^* transgenic *Arabidopsis* plants was extracted using plant RNA extraction kit (E.Z.N.A.^®^Plant RNA Kit, OMEGA), and the first strand cDNA was synthesized using Oligotex-dT30 (TaKaRa). RT-PCR was performed to determine the expression of *BnAHAS1^544T^*, with the housekeeping gene *Ubiquitin-conjugating enzyme 21* (*UBC21*, At5g25760) as the internal reference. A primer pair A1-forward (5′_ATCCCCTCTACCCATTTCC_3′) and A1-reverse (5′_TTGTCGGTTTTTTCAGGGG_3′) was used for amplification of *BnAHAS1^544T^* and a primer pair UBC21-forward (5′_CTGCGACTCAGGGAATCTTCTAA_3′) and UBC21-reverse (5′_TTGTGCCATTGAATTGAACCC_3′) was used for amplification of *UBC21*.

### Enzyme Extraction and AHAS Activity Determination of Rapeseed

Rapeseed AHAS enzyme was extracted and detected according to the protocols described by Singh et al. (1988) and Sibony et al. (2001). In brief, each sample of cauline leaf or lateral inflorescence was collected from 10 rapeseed plants at each experimental plot mentioned above 10 days after TBM treatment, and three replications were included for each sample. One gram of each sample was ground to powder on ice. Crude enzyme was extracted using 5 ml of extraction buffer [100 mM potassium phosphate buffer (pH = 7.0), 1 mM sodium pyruvate, 0.5 mM MgCl_2_, 0.5 mM thiamine pyrophosphate (TPP), 10 μM flavine adenine dinucleotide (FAD)], and dissolved in 5 ml elution buffer [100 mM potassium phosphate buffer (pH = 7.0), 20 mM sodium pyruvate, 0.5 mM MgCl_2_]. The activity of AHAS enzyme was determined by measuring the amount of acetolactate produced in 2 ml volume containing 1 ml enzyme solution and 1 ml reaction buffer [100 mM potassium phosphate buffer (pH = 7.0), 20 mM sodium pyruvate, 0.5 mM MgCl_2_, 0.5 mM thiamine pyrophosphate, 10 μM flavine adenine dinucleotide]. After decarboxylated by 3 M sulfuric acid, the colored products formed by the reaction of acetoin and creatine and α-naphthol were measured using absorbance at 525 nm on Spectroscopy (Westerfeld, 1945). One unit of AHAS activity (1U) was defined as the amount of AHAS required for producing 1 mg acetolactate in an hour. The relative activity of AHAS was described as the percentage of absorbance value of each sample of TBM-treated plants and the corresponding sample of the control plants (treated with 0 mg⋅L^-1^ of TBM).

### Agronomic Traits

Agronomic traits of rapeseed plants treated with different concentrations of TBM solution were evaluated at maturity. Ten plants in each experimental plot were randomly selected for assessing the following 10 agronomic traits: plant height, setting position of the first primary branch, the number of primary branches per plant, terminal raceme length, the number of siliques on the terminal raceme, the number of siliques per plant, seed number per silique, silique length, thousand-seed weight, seed yield per plant. Three traits, early flowering period, final flowering period, and mature period were also recorded for each plot.

### Data Analysis

In order to reveal the correlation between the percentage of sterile plants and the relative AHAS activity, we used a modified logistic function, *Y* = 100-*A*/(1+*Be*^(-^*^KX^*^)^), whereas, *Y* represents the percentage of sterile plants; *X* indicates the relative activity of AHAS as described above; *A, B*, and *K* stand for the constants in logistic function that were estimated by the method proposed by Dong (2007) using SPSS 19.0 software (SPSS Inc., 2010). The goodness-of-fit test was evaluated by calculating pseudo-*R*^2^ (Juneja et al., 2007). The AHAS activity value at the inflection point was calculated from the modified logistic equations when *Y* = *A*/2.

For variance analysis of the tested traits, including the percentage of male sterile, AHAS activity, and agronomic traits, a randomized complete block design was used by SPSS 19.0 software (SPSS Inc., 2010).

## Results

### Agronomic Performance of the Mutant Line *K5*

Main agronomic traits of the mutant line *K5* and wild type *ZS9* were investigated and compared for evaluating the utilization potentiality of the mutant line. Compared with *ZS9*, the early flowering time of *K5* was postponed for 8 days, and the growth period was extended for 5 days more. The number of primary branches per plant and the number of siliques per plant of *K5* were greater than those of *ZS9*, while the number of siliques in the terminal raceme, the number of seeds per silique, silique length, and thousand-seed weight of *K5* were less than those of *ZS9* (**Table 1**). But finally, there was no significant difference in seed yield per plant between *K5* and *ZS9* (**Table 1**).

**Table 1:** Comparison of main agronomic traits of *ZS9* and the mutant line *K5* in *Brassica napus* L.

Line	EFP (d)	FFP (d)	MP (d)	PH (cm)	SPFPB (cm)	NB	LTR (cm)	NSTR	NSP	SL (cm)	Seed no.	TSW (g)	SYP (g)
*ZS9*	183.0 ± 0.8	210.0 ± 1.0	244.0 ± 1.0	146.7 ± 6.0	39.1 ± 8.3	9.5 ± 1.1	57.5 ± 4.7	78.2 ± 8.0	374.7 ± 91.9	6.8 ± 0.2	19.3 ± 4.0	3.2 ± 0.2	20.3 ± 7.4
*K5*	191.0 ± 1.0^∗^	220.0 ± 0.8^∗^	249.0 ± 0.0^∗^	141.5 ± 11.1	41.6 ± 9.1	11.6 ± 1.7^∗^	51.6 ± 7.7	63.5 ± 20.9^∗^	533.1 ± 166.7^∗^	5.7 ± 0.6^∗^	14.3 ± 1.5^∗^	2.6 ± 0.5^∗^	18.4 ± 11.4

*EFP, early flowering period; FFP, final flowering period; MP, mature period; PH, plant height; SPFPB, setting position of the first primary branch; NB, the number of primary branches per plant; LTR, length of the terminal raceme; NSTR, the number of siliques on the terminal raceme; NSP, number of siliques per plant; SL, silique length; Seed no., seed numbers per silique; TSW, thousand seed weight; SYP, seed yield per plant. Data in the table are expressed by mean ± SD, n = 6. “^∗^”: meanssignificant difference at P = 0.05 level between both lines*.

### Identification of Mutation in *BnAHAS* Genes in *K5*

**FIGURE 1:**
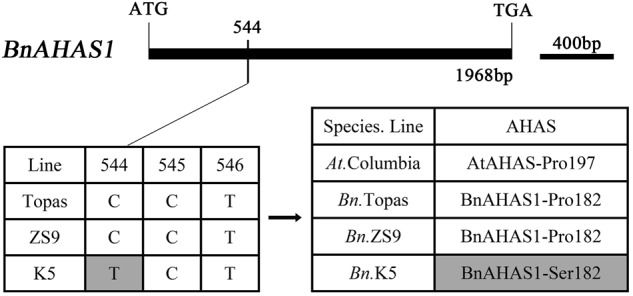
Comparisons of DNA and protein of *BnAHAS1* from *Topas, ZS9* and *K5* in *Brassica napus* L. The sequence of *BnAHAS1* of *Topas* is from NCBI (GenBank ID: Z11524.1). AtAHAS was regarded as the reference protein.

There are three functional *AHAS* genes in rapeseed (*B. napus*), *BnAHAS1, BnAHAS2*, and *BnAHAS3* (Rutledge et al., 1991; Ouellet et al., 1992). In order to determine whether there is any mutations in the *BnAHAS* sequences of *K5*, the *BnAHAS1, BnAHAS2*, and *BnAHAS3* of *K5* and *ZS9* were all cloned and sequenced. As a result, no nucleotide difference in the coding region of *BnAHAS2* and *BnAHAS3* were identified between *K5* and *ZS9* (data not shown). But a nucleotide mutation was detected in the *BnAHAS1* of *K5*, i.e., a cytidylate at position 544 from the translation start site was replaced by a thymidylate, compared with that of *ZS9* and *Topas* (a rapeseed variety, whose *AHAS* sequences were registered in Genbank), and this mutant allele was designed as *BnAHAS1^544T^*, whose sequence was deposited in the GenBank (accession number: KP985786). This mutation in *BnAHAS1* of *K5* resulted in the substitution of 182 amino acids (Pro to Ser) in BnAHAS1, which corresponds to Pro-197 in AtAHAS, therefore it was named as Pro-182-Ser in BnAHAS1 (**Figure 1**).

### TBM-Treatment Could Induce Male Sterility in Both *ZS9* and *K5*

**FIGURE 2:**
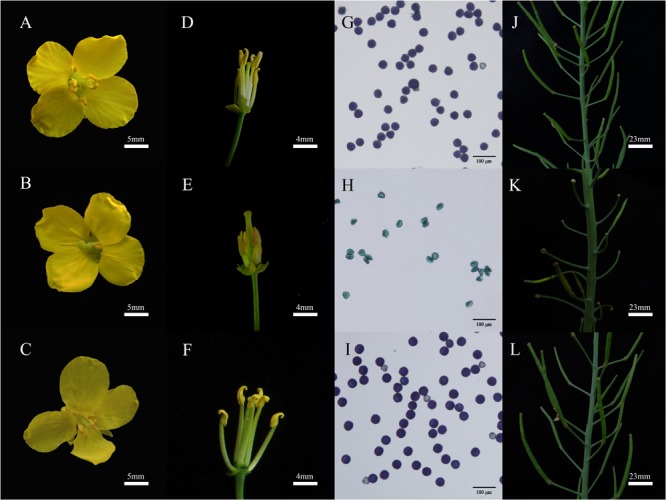
Phenotype of rapeseed *ZS9* and mutant line *K5* treated with tribenuron-methly (TBM). **(A,D)** Photograph of flower from *ZS9* plants treated with 0 mg⋅L^-1^ TBM. **(B,E)** Photograph of flower from *ZS9* plants treated with 0.10 mg⋅L^-1^ TBM. **(C,F)** Photograph of flower from *K5* plants treated with 0.10 mg⋅L^-1^ TBM. **(G–I)** Pollens viability of *ZS9* plants treated with 0 mg⋅L^-1^ TBM **(G)** and with 0.10 mg⋅L^-1^ TBM **(H)**, and *K5* plants treated with 0.10 mg⋅L^-1^ TBM **(I)**, respectively (bars = 100 μm). **(J–L)** Seed setting of *ZS9* plants with 0 mg⋅L^-1^ TBM **(J)**, *ZS9* **(K)**, and *K5* **(L)** plants treated with 0.10 mg⋅L^-1^ TBM by bag-selfing, respectively.

Five concentrations of TBM solution (see section “Materials and Methods”) were applied to *ZS9* plants at the bolting stage through foliar-spraying. Analysis of variance (ANOVA) showed that there were significant differences in their male sterility-inducing abilities among different treatments. The 0.05 mg⋅L^-1^ TBM-treatment could induce 7.5% male sterility, while 0.10 mg⋅L^-1^ TBM-treatment or above could induce 96.7% or higher male sterility (**Table 2**). Observation of the floral morphological characteristics of *ZS9* plants treated with 0.10 mg⋅L^-1^ TBM and the control *ZS9* plants (treated with 0 mg⋅L^-1^ of TBM) found that unlike the normal flowers in the control plants (**Figures 2A,D**), stamen of male sterile flowers were below the corolla and could barely be seen from outside due to remarkable elongation-inhibited filaments (**Figures 2B,E**). Moreover, unlike the pollens in the control plants (**Figure 2G** and **Table 2**), almost no fertile pollens were observed in the *ZS9* plants treated with 0.10 mg⋅L^-1^ TBM (**Figure 2H** and **Table 2**). There is no seed-setting upon bag-selfing (**Figure 2K**), but seed-setting recover normal upon open pollination (data not shown). Furthermore, the agronomic traits of *ZS9* plants treated with different concentrations of TBM were investigated at maturity stage. The results indicated that no significant difference was detected in these agronomic traits between control plants and the plants treated with TBM solution ≤0.10 mg⋅L^-1^ (**Table 3**). However, 1.00 and 2.00 mg⋅L^-1^ TBM-treatments could induce complete male sterility of *ZS9* (**Table 2**) and lead to significant decrease in most of the yield-related traits tested, such as silique length, seed number per silique, and thousand- seed weight, etc. (**Table 3** and **Supplementary Figure S1**). These results suggested that the application of 5 ml of 0.10 mg⋅L^-1^ TBM to per rapeseed *ZS9* plant could meet the requirement of CHA that can induce male sterility without significantly phytotoxic effects on agronomic traits and seed yield, this is consistent with the previous results (Yu et al., 2006; Zhang et al., 2010; Li H. et al., 2015; Zhao et al., 2015).

**Table 2:** Comparison of male sterility of *ZS9* and the mutant line *K5* treated with different concentrations of tribenuron-methly (TBM).

TBM (mg⋅L^-1^)	*ZS9*		*K5*	
	Percent of sterile pollens (%)	Percent of sterile plants (%)	Percent of sterile pollens (%)	Percent of sterile plants (%)
0	4.8 ± 3.0c	0.0 ± 0.0d	7.9 ± 1.7d	0.0 ± 0.0e
0.05	67. 7 ± 6.9b	7.5 ± 2.3c	8.2 ± 1.1d	0.0 ± 0.0e
0.10	99.9 ± 0.3a	96.7 ± 3.1b	8.5 ± 1.9d	0.0 ± 0.01e
1.00	100.0 ± 0.0a	100.0 ± 0.0a	7.8 ± 1.5d	1.0 ± 0.6d
2.00	100.0 ± 0.0a	100.0 ± 0.0a	9.0 ± 1.9d	2.7 ± 1.3d
5.00	–	–	12.0 ± 2.7d	4.1 ± 1.9d
10.00	–	–	47.5 ± 4.8c	37.5 ± 5.9c
15.00	–	–	68.1 ± 7.2b	58.2 ± 6.1b
20.00	–	–	98.2 ± 0.5a	100 ± 0.0a
30.00	–	–	98.7 ± 1.1a	100 ± 0.0a
40.00	–	–	99.4 ± 0.6a	100 ± 0.0a

*Data in the table are expressed by mean ± SD, n = 6. Data followed by different superscript lowercase letter within same column means significant difference at P = 0.05 level. “–”: not available.*

**Table 3:** Main agronomic traits of *ZS9* treated with different concentrations of tribenuron-methly (TBM).

TBM (mg⋅L^-1^)	PH (cm)	SPFPB (cm)	NB	LTR (cm)	NSTR	NSP	SL (cm)	Seed no.	TSW (g)	SYP (g)
0	146.7 ± 6.0^a^	39.1 ± 8.3^a^	9.5 ± 1.1^b^	57.5 ± 4.7^a^	78.2 ± 8.0^a^	374.7 ± 91.9^a^	6.8 ± 0.2^a^	19.9 ± 4.1^a^	3.2 ± 0.2^a^	20.3 ± 7.4^a^
0.05	143.5 ± 11.6^a^	35.6 ± 13.4^ab^	9.6 ± 1.9^ab^	54.2 ± 8.2^a^	72.9 ± 10.7^a^	377.9 ± 121.7^a^	7.0 ± 0.4^a^	21.0 ± 3.6^a^	3.1 ± 0.5^a^	19.3 ± 5.2^a^
0.10	147.3 ± 5.1^a^	35.2 ± 10.5^ab^	9.8 ± 1.3^ab^	58.8 ± 7.2^a^	77.6 ± 7.6^a^	372.6 ± 66.4^a^	7.0 ± 0.4^a^	22.6 ± 2.6^a^	3.3 ± 0.5^a^	22.3 ± 6.5^a^
1.00	120.8 ± 9.3^b^	29.8 ± 10.8^b^	10.6 ± 1.3^a^	30.4 ± 10.1^b^	26.0 ± 15.1^b^	406.9 ± 116.4^a^	5.3 ± 0.3^b^	17.0 ± 4.6^b^	2.8 ± 0.3^b^	17.4 ± 6.0^a^
2.00	95.3 ± 7.7^c^	18.7 ± 12.1^c^	10.5 ± 2.6^a^	12.5 ± 8.6^c^	9.5 ± 7.7^c^	428.4 ± 193.7^a^	4.1 ± 0.4^c^	11.7 ± 1.9^c^	2.3 ± 0.4^c^	9.8 ± 9.1^b^

*PH, plant height; SPFPB, setting position of the first primary branch; NB, the number of primary branches per plant; LTR, length of the terminal raceme; NSTR, the number of siliques on the terminal raceme; NSP, number of siliques per plant; SL, silique length; Seed no., seed numbers per silique; TSW, thousand seed weight; SYP, seed yield per plant. Data in the table are expressed by mean ± SD, n = 6. Data followed by different superscript lowercase letter within same column means significant difference at P = 0.05 level*.

In order to investigate whether the mutant line *K5* can also be induced male sterility, 11 concentrations of TBM solution (see section “Materials and Methods”) were applied to *K5* plants at the bolting stage through foliar-spraying. ANOVA showed that there were significant differences in their male sterility-inducing abilities among different treatments. The 0.05 and 0.10 mg⋅L^-1^ TBM-treatments could not induce male sterility in *K5* plants; five TBM-treatments (1.00, 2.00, 5.00, 10.00, and 15.00 mg⋅L^-1^) could induce partial male sterility of *K5* plants; and 20.00 mg⋅L^-1^ and above TBM-treatments could induce complete male sterility of *K5* plants (**Table 2** and **Supplementary Figure S2**). Male reproductive organs of *K5* plants showed normal under 0.10 mg⋅L^-1^ TBM treatment (**Figures 2C,F,I,L**), but this TBM treatment could induce complete male sterility of rapeseed *ZS9*. We also investigated agronomic traits of the control (treated with 0 mg⋅L^-1^ of TBM) and TBM-treated plants of *K5* at maturity stage. The results showed that under the treatment of 0.10 mg⋅L^-1^ TBM, no significant differences were detected in all agronomic traits between the control plants and the TBM-treated plants of *K5*, while, under the treatment of 20.00 mg⋅L^-1^ TBM, no significant differences were detected in all the agronomic traits except for four traits (length of the terminal raceme, the number of siliques on the terminal raceme, silique length, seed numbers per silique) between the control plants and the TBM-treated plants (**Table 4**).

**Table 4:** Main agronomic traits of the mutant line *K5* treated with different concentrations of tribenuron-methly (TBM).

TBM (mg⋅L^-1^)	PH (cm)	SPFPB (cm)	NB	LTR (cm)	NSTR	NSP	SL (cm)	Seed no.	TSW (g)	SYP (g)
0	141.5 ± 11.1^a^	41.6 ± 9.1^a^	10.6 ± 1.2^b^	51.6 ± 7.7^a^	63.5 ± 20.9^a^	533.1 ± 166.7^a^	5.7 ± 0.6^a^	14.3 ± 1.5^a^	2.6 ± 0.5^a^	18.4 ± 11.4^a^
0.05	146.7 ± 12.9^a^	46.7 ± 11.7^a^	10.7 ± 1.8^b^	54.2 ± 10. 4^a^	64.2 ± 13.0^a^	469.6 ± 152.0^a^	5.7 ± 0.6^a^	15.2 ± 2.1^a^	2.8 ± 0.4^a^	17.6 ± 8.3^a^
0.10	142.8 ± 14.4^a^	44.7 ± 10.5^a^	10.8 ± 1.2^b^	52.1 ± 9.0^a^	65.9 ± 12.6^a^	466.9 ± 110.6^a^	5.8 ± 0.5^a^	14.1 ± 2^a^	2.8 ± 0.2^a^	17.8 ± 8.0^a^
1.00	151.8 ± 14.2^a^	43.1 ± 12.4^a^	11.6 ± 1.1^ab^	57.5 ± 7.3^a^	72.4 ± 12.1^a^	518.8 ± 129.5^a^	5.7 ± 0.4^a^	13.9 ± 2.3^a^	2.7 ± 0.3^a^	19.7 ± 7.0^a^
2.00	152.2 ± 10.4^a^	43.1 ± 9.2^a^	12.1 ± 1.8^a^	56.9 ± 9.4^a^	70.7 ± 10.8^a^	574.7 ± 130.7^a^	5.8 ± 0.5^a^	13.6 ± 2.3^a^	2.8 ± 0.4^a^	24.5 ± 6.3^a^
5.00	147.4 ± 11.3^a^	37.1 ± 10.6^a^	12.2 ± 1.5^a^	54.7 ± 8.1^a^	62.9 ± 15.2^a^	605.4 ± 175.6^a^	5.7 ± 0.7^a^	13.7 ± 3.3^a^	2.8 ± 0.4^a^	21.8 ± 10.4^a^
10.00	148.7 ± 9.9^a^	40.7 ± 14.0^a^	12.3 ± 1.7^a^	54.6 ± 6.6^a^	68.3 ± 8.6^a^	583.3 ± 230.2^a^	5.7 ± 0.5^a^	14.2 ± 3.1^a^	2.8 ± 0.3^a^	24.4 ± 12.1^a^
15.00	149.7 + 25.7^a^	39.0 + 10.0^a^	13.3 + 1.5^a^	52.3 + 16.1^a^	59.0 + 6.6^a^	459.7 + 70.4^a^	5.2 + 0.6^a^	13.9 + 1.8^a^	2.8 ± 0.4^a^	19.9 ± 7.8^a^
20.00	131.3 + 17.1^a^	42.8 + 9.1^a^	12.5 + 0.6^a^	35.3 + 9.8^b^	35.8 + 4.9^b^	507.5 + 163.1^a^	3.1 + 0.7^b^	9.6 + 3.8^b^	2.7 ± 0.5^a^	15.1 ± 4.6^ab^
30.00	125.0 + 9.6^a^	38.3 + 2.5^a^	12.7 + 1.2^a^	24.7 + 4.0^b^	11.0 + 1.0^c^	323.7 + 104.4^ab^	3.1 + 0.9^b^	8.2 + 1.7^b^	2.4 ± 0.4^ab^	9.4 ± 6.4^ab^
40.00	112.3 + 12.7^b^	29.0 + 12.2^a^	10.7 + 0.6^b^	24.0 + 4.4^b^	9.0 + 5.6^c^	168.3 + 73.9^b^	2.6 + 0.7^b^	6.6 + 1.7^b^	1.7 ± 0.5^b^	6.2 ± 4.7^b^

*PH, plant height; SPFPB, setting position of the first primary branch; NB, the number of primary branches per plant; LTR, length of the terminal raceme; NSTR, the number of siliques on the terminal raceme; NSP, number of siliques per plant; SL, silique length; Seed no., seed numbers per silique; TSW, thousand seed weight; SYP, seed yield per plant. Data in the table are expressed by mean ± SD, n = 6. Data followed by different superscript lowercase letter within same column means significant difference at P = 0.05 level*.

Taken together, the application of 5 ml of 0.10 mg⋅L^-1^ TBM to per plant of *ZS9* can induce male sterility, without significantly phytotoxic effects on agronomic traits and seed yield. The TBM-tolerance of *K5* male reproductive organs was approximately 200 times that of *ZS9*.

### The Relationship Between TBM-Induced Male Sterility and the Relative Activity of AHAS in Both *ZS9* and *K5*

**FIGURE 3:**
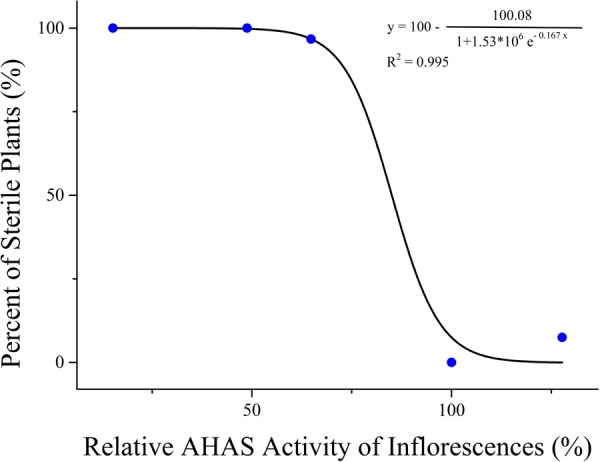
Correlation analysis of inflorescence AHAS activity and male sterility in rapeseed *ZS9*.

To reveal the relationship between the changes of AHAS activity and the TBM-induced male sterility, AHAS activity of leaves and inflorescences in rapeseed plants was detected at the 10th day after TBM-treatment. The results showed significant differences in AHAS activity of leaves and inflorescences of *ZS9* plants among different TBM treatments (**Table 5**). The treatment of 0.05 mg⋅L^-1^ TBM could increase AHAS activity of leaves and inflorescences of *ZS9* plants (**Table 5**). The treatment of 0.10 mg⋅L^-1^ TBM did not significantly affect AHAS activity of leaves of *ZS9*, but could significantly decrease AHAS activity of inflorescence (**Table 5**), and induced nearly complete male sterility of *ZS9* plants (**Table 2**). It seemed that there was not any clear relationship between the changes of AHAS activity of leaves and TBM-induced male sterility of *ZS9*. We further used the logistic function to explore the relationship between the percentage of sterile plants and the relative AHAS activity of inflorescences of TBM-treated *ZS9* plants. A modified logistic function, *Y* = 100-100.08/(1+1.53^∗^10^6^e^(-0.167^*^X^*^)^), could best fit the observed data (pseudo-*R*^2^ = 0.995), where, *Y* represents the percentage of sterile plants; *X* indicates the relative activity of AHAS of inflorescences (**Figure 3**). The value of AHAS activity at the inflection point was 85.27%, whereas *Y* = 50.04% calculated from the modified logistic equation. However, the percentage of sterile plants did not correlate with the relative activity of AHAS of leaves of TBM-treated *ZS9* plants (data not shown).

**Table 5:** Changes of AHAS activity of *ZS9* treated with different concentrations of tribenuron-methly (TBM)^#^.

TBM (mg⋅L^-1^)	AHAS activity of leaves		AHAS activity of inflorescences	
	Specific activity (U/mg protein)	Relative activity (%)^∗^	Specific activity (U/mg protein)	Relative activity (%)^∗^
0	0.389 ± 0.05B	100.000 ± 12.078B	0.892 ± 0.102A	100.000 ± 11.422A
0.05	0.490 ± 0.02A	125.966 ± 5.020A	1.112 ± 0.037A	127.715 ± 4.204A
0.10	0.432 ± 0.03AB	110.855 ± 7.660AB	0.566 ± 0.086B	64.819 ± 13.175B
1.00	0.147 ± 0.01C	37.807 ± 2.982C	0.466 ± 0.043B	48.778 ± 4.869B
2.00	0.116 ± 0.02C	29.822 ± 5.124C	0.139 ± 0.037C	15.177 ± 5.843C

*^#^Data in the table are expressed by mean ± SD, n = 6. Data followed by different superscript capital letter within same column means significant difference at P = 0.01 level. ^∗^Relative activity of AHAS was determined as the percentage of absorbance value of each sample of TBM-treated plants and the corresponding sample of the control plants (treated with 0 mg⋅L^*-1*^ of TBM).*

No significant difference was detected in AHAS activities of leaves or inflorescences between *ZS9* and *K5* without TBM treatment (**Tables 5, 6** and **Supplementary Figure S3**). Remarkably, great difference was tested in AHAS activities between the leaves and the inflorescences in each of the two genotypes, where the AHAS activity of inflorescences was significantly higher than that of leaves in both genotypes (**Tables 5, 6** and **Supplementary Figure S3**).

**Table 6:** Changes of AHAS activity in the mutant line *K5* treated with different concentrations of tribenuron-methly (TBM)^#^.

TBM (mg⋅L^-1^)	AHAS activity of leaves		AHAS activity of inflorescences	
	Specific activity (U/mg protein)	Relative activity (%)^∗^	Specific activity (U/mg protein)	Relative activity (%)^∗^
	0.360 ± 0.119B	100.000 ± 13.179B	0.715 ± 0.089A	100.000 ± 12.479A
0.05	0.705 ± 0.069A	195.774 ± 19.185A	0.680 ± 0.083A	98.759 ± 11.587A
0.10	0.534 ± 0.059AB	148.304 ± 16.517AB	0.637 ± 0.068AB	89.162 ± 9.535AB
1.00	0.519 ± 0.100AB	154.380 ± 30.551AB	0.566 ± 0.033BC	79.245 ± 4.654BC
2.00	0.524 ± 0.097AB	145.648 ± 26.895AB	0.530 ± 0.055BC	74.139 ± 7.688BC
5.00	0.371 ± 0.158B	91.991 ± 11.888B	0.472 ± 0.101CD	67.818 ± 14.993CD
10.00	0.330 ± 0.168B	91.584 ± 22.579B	0.406 ± 0.023D	56.802 ± 3.286D
15.00	0.291 ± 0.022B	80.724 ± 6.204B	0.299 ± 0.019E	41.828 ± 2.620E
20.00	0.206 ± 0.053C	57.117 ± 14.852C	0.237 ± 0.044EF	33.213 ± 6.094EF
30.00	0.194 ± 0.047C	53.977 ± 11.791C	0.185 ± 0.042FG	25.958 ± 5.922FG
40.00	0.149 ± 0.032C	41.291 ± 9.016C	0.157 ± 0.020GH	21.948 ± 2.647GH

*^#^Data in the table are expressed by mean ± SD, n = 6. Data followed by different superscript capital letter within same column means significant difference at P = 0.01 level. ^∗^Relative activity of AHAS was determined as the percentage of absorbance value of each sample of TBM-treated plants and the corresponding sample of the control plants (treated with 0 mg⋅L^-1^ of TBM).*

**FIGURE 4:**
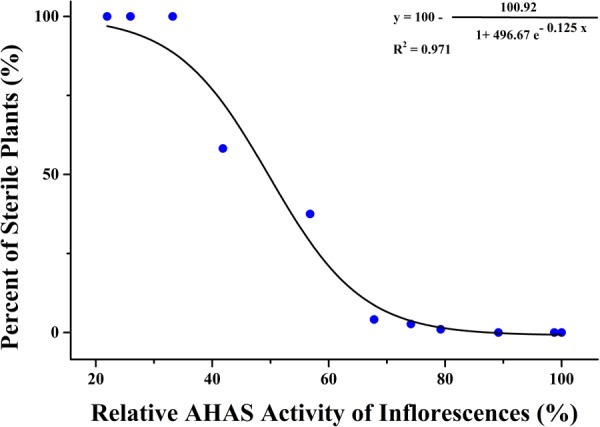
Correlation analysis of inflorescence AHAS activity and male sterility in mutant line *K5*.

Significant differences were tested in AHAS activity of leaves and inflorescences of *K5* plants among different TBM treatments (**Table 6**). The treatment of 0.05 mg⋅L^-1^ TBM significantly increase the AHAS activity of the leaves of *K5* plants, but did not affect the AHAS activity of the inflorescences; while other six TBM-treatments (0.10, 1.00, 2.00, 5.00, 10.00, and 15.00 mg⋅L^-1^) did not significantly affect the AHAS activity of leaves of *K5* plants, compared with control (0 mg⋅L^-1^ TBM treatment), but all these treatments significantly decreased the AHAS activity of the inflorescences except for the 0.10 mg⋅L^-1^ TBM-treatment; the treatments of 20.00 mg⋅L^-1^ or higher concentration TBM significantly decreased the AHAS activity of both the leaves and inflorescences of *K5* plants (**Table 6**). Generally, the AHAS activity of the inflorescences of *K5* plants gradually decreased with the increase of TBM concentration (**Table 6**). We also used the modified logistic function mentioned above to show the relationship between the percentage of sterile plants and the relative activity of AHAS of the inflorescences of *K5* plants. A modified logistic function, *Y* = 100-100.92/(1+496.67e^(-0.125^*^X^*^)^), could best fit the observed data (pseudo-*R*^2^ = 0.971), where, *Y* indicates the percentage of sterile plants; *X* represents the relative activity of AHAS of the inflorescences *K5* plants. The value of AHAS activity at the inflection point was 49.51%, where *Y* = 50.02% (**Figure 4**).

### Transgenic Confirmation of *BnAHAS1^544T^* as the Causal Gene for the Increased TBM-Resistance of Male Organs in Mutant Line *K5*

**FIGURE 5:**
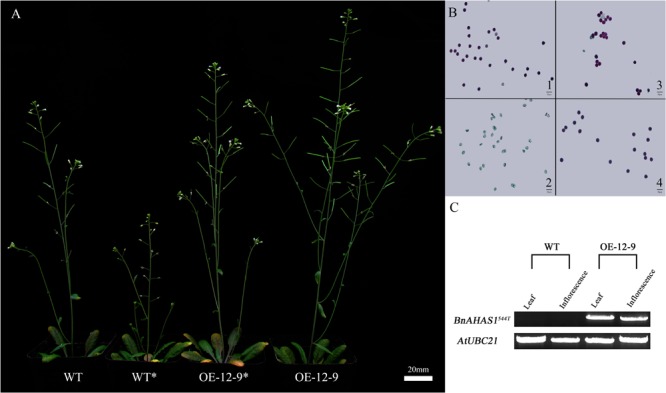
Over-expression of *BnAHAS1^544T^* increases TBM-resistance in *Arabidopsis*. **(A)** Phenotypes of plants treated with 0 and 0.007 mg⋅L^-1^ tribenuron-methly (TBM). WT and WT^∗^ represent wild type plants treated with 0 and 0.007 mg⋅L^-1^ TBM, respectively. OE-12-9 and OE-12-9^∗^ indicate transgenic lines treated with 0 and 0.007 mg⋅L^-1^ TBM, respectively. **(B)** Pollens viability of *Arabidopsis* plants. 1 and 2, wild type plants treated with 0 and 0.007 mg⋅L^-1^ TBM, respectively; 3 and 4, transgenic line OE-12-9 treated with 0 and 0.007 mg⋅L^-1^ TBM, respectively; bars = 50 μm. **(C)** Over-expression level of *BnAHAS1^544T^* in WT and OE-12-9 plants.

In order to confirm whether the higher TBM-resistance of male reproductive organs of the mutant line *K5* was conferred by *BnAHAS1^544T^*, this allele was introduced into *A. thaliana* to develop transgenic plants. As a result, 16 transgenic events were generated. Three homozygous transgenic lines *35s::BnAHAS1^544T^*-12-9, *35s::BnAHAS1^544T^*-1-4, and *35s::BnAHAS1^544T^*-3-10 (hereinafter refer as OE-12-9, OE-1-4, and OE-3-10, respectively, for short) were obtained by screening and selfing, and employed for TBM-resistance assessment. First, we did pre-experiment by foliar-spraying different concentration of TBM (0, 0.004 and 0.007 mg⋅L^-1^) to wild type *A. thaliana*, and found that 0.007 mg⋅L^-1^ TBM can induce male sterility (**Supplementary Figure S4**). Then, we treated the three transgenic lines (OE-12-9, OE-1-4, and OE-3-10) and wild type with 0 and 0.007 mg⋅L^-1^ TBM, respectively, through foliar-spraying at the bolting stage. The phenotypes of the transgenic line OE-12-9 treated or untreated were shown in **Figure 5A** as a representative. Pollens from the three transgenic lines treated with 0.007 mg⋅L^-1^ TBM exhibited good viability, while pollens from wild type plants treated with 0.007 mg⋅L^-1^ TBM showed no viability (**Figure 5B**). RT-PCR analysis indicated that *BnAHAS1^544T^* was expressed in the transgenic line OE-12-9, but not in wild type plants (**Figure 5C**). These results indicated that ectopic expression of *BnAHAS1^544T^* increased TBM-tolerance of male reproductive organs of *A. thaliana.* Together with the fact that rapeseed mutant line *K5* with *BnAHAS1^544T^* allele exhibits a higher TBM-resistance of male reproductive organs, compared with its wild type, we can conclude that a higher TBM-resistance of male reproductive organs in the mutant line *K5* should be attributed to *BnAHAS1^544T^*.

**FIGURE 6:**
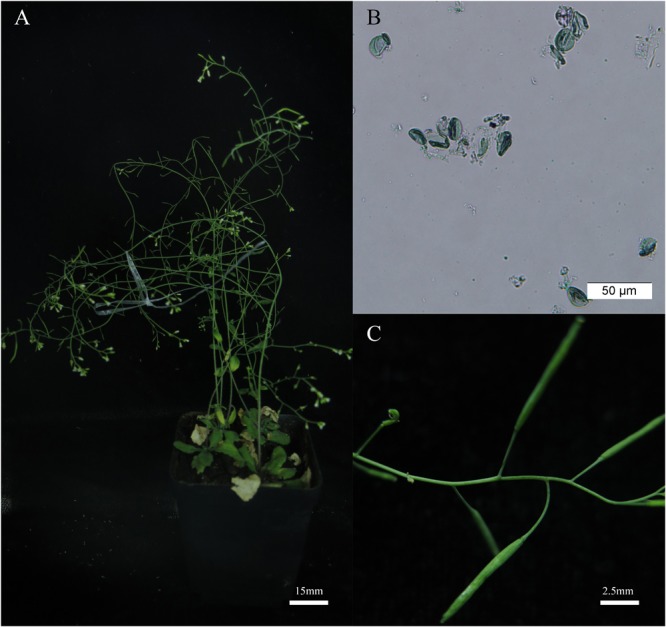
The *BnAHAS1^544T^* transgenic *Arabidopsis* plants showed resistance and pistil fertility under 2.00 mg⋅L^-1^ tribenuron-methly (TBM) treatment. **(A)** Transgenic plants (OE-1-4) treated with 2.00 mg⋅L^-1^ TBM. **(B)** Pollens viability of the transgenic plants shown in **(A)**. **(C)** Pods can be obtained by artificial pollination of transgenic plants after treated with 2.00 mg⋅L^-1^ TBM.

Further experiments showed that the *BnAHAS1^544T^* transgenic lines could also be induced to male sterility under treatment of 2 mg⋅L^-1^ TBM (**Figure 6**), this concentration of TBM was above the lethal dose for *A. thaliana* wild type (0.1 mg⋅L^-1^, **Supplementary Figure S5**).

## Discussion

### A Novel TBM-Resistant Mutant *K5* Is a Valuable Resource for Rapeseed Breeding

Herbicide resistant plants can provide not only an economic way to control weeds in crop production, but also a new way to utilize the heterosis. CIMS system is an important pollination control system for utilization of heterosis in rapeseed. However, in hybrid seed production, male parents, normal varieties without resistant to the CHAs, must be protected with a shield to avoid it stamens being injured when spraying CHAs to female parents to induce male sterility, this not only complicate the seed production system and but also increase the cost of hybrid seed production. Li H. et al. (2015) proposed a strategy of utilizing herbicide-resistant line as a male parent, which would simplified the hybrid seed production using the CIMS system. In the present study, the rapeseed *ZS9* showed nearly complete male sterility without obviously adverse effects on main agronomical traits when treated with 0.1 mg⋅L^-1^ TBM, however, the mutant line *K5* exhibited normal male fertility and its agronomical traits were not significantly affected under the same TBM concentration (**Figure 2** and **Tables 2, 3**). The mutant line *K5* could be induced complete male sterility when TBM concentration was increased to 200 mg⋅L^-1^ that is approximately 200 times that of ZS9. Our previous investigation revealed that foliar-spraying with 2 mg⋅L^-1^ TBM at the seedling stage killed all the 49 genotypes of *B. napus* tested (Xin et al., 2014). So, the *K5* line is a valuable resource for rapeseed hybrid breeding by CIMS approach.

There are three functional *AHAS* genes in rapeseed (*B. napus*), *BnAHAS1, BnAHAS2*, and *BnAHAS3* (Rutledge et al., 1991; Ouellet et al., 1992). In the present study, sequencing analysis of the three functional *AHAS* genes in the *K5* only revealed a mutation in *BnAHAS1*, i.e., a cytidylate replaced by a thymidylate at position 544 from the translation start site in the *K5* (this allele named as *BnAHAS1^544T^*), comparing with that of *ZS9*. This mutation resulted in an amino acid replacement (Pro to Ser) at position 182 in the BnAHAS1 (Pro197 in reference to *Arabidopsis*). To the best of our knowledge, *BnAHAS1^544T^* was a novel resistant allele that has not been reported in rapeseed previously (Swanson et al., 1989; Tan et al., 2005; Hu et al., 2015, 2017; Li H. et al., 2015).

In the previous studies, the first two microspore-derived rapeseed mutants (PM1 and PM2) were similar to the parent Topas for yield, maturity, disease resistance and oil quality (Swanson et al., 1989). Maltais and Bouchard (1978) reported that transferring a spontaneous atrazine-tolerant *B. rapa* mutant trait into canola generated a canola cultivar ‘Triton.’ However, the yield of this canola cultivar was only 80% of that normal canola cultivars and its oil was almost 2.5% less (Beversdorf and Hume, 1984). Magha et al. (1993) reported that a spontaneous rapeseed mutant tolerant to sulfonylurea and imidazoline herbicides showed less productive and 1 week later than sensitive parent. The present study showed that there was no significant difference between the mutant line *K5* and *ZS9* in seed yield per plant without TBM treatment, however, *K5* delayed its flowering time for 8 days and extended its growth period for 5 days, compared with wild type *ZS9*. Besides the mutation of *BnAHAS1^544T^*, there may be some other mutated sites in the *K5* which affect its growth period traits, but they do not contribute to the higher resistance to TBM, as our genetic analysis indicated that the higher TBM resistance of the *K5* line was controlled by a single dominant nuclear gene, and allele-specific markers for detecting the C-544-T variation in *BnAHAS1* we developed were co-segregated with the herbicide resistance in rapeseed (not published). For the practical utilization of *K5*, we have improved it through crossing, back-crossing and marker-assisted selection (based on allele-specific markers for detecting the C-544-T variation in *BnAHAS1*) and obtained some elite lines with early flowering and maturity traits compared with *K5*.

### The Potential Target of TBM When Used as CHA

It has been confirmed that AHAS should be the target of AHAS-inhibiting herbicides (Pang et al., 2003; Mccourt et al., 2005, 2006). Recently, a few SU-herbicides were found to be excellent CHAs for rapeseed at a lower concentration (Yu et al., 2006, 2009, 2017; Cheng et al., 2013, 2015; Yu and He, 2014), which attracted many researchers to reveal its underlying mechanism using different approaches (Cheng et al., 2013; Li Z. et al., 2015; Zhao et al., 2015; Liu et al., 2017). Transcriptional and proteomic analyses have identified many differential expression transcripts and proteins in monosulfuron-ester sodium- or amidosulfuron-induced male sterile rapeseed (Cheng et al., 2013; Li Z. et al., 2015; Liu et al., 2017). Zhao et al. (2015) suggested that AHAS is the sole target of TBM when used as a CHA based on a comprehensive analysis of cytology, pharmacology and transgene, and they revealed the relationship between AHAS activity and male sterility by using of *Arabidopsis* mutant *csr1-1D* and *csr1-1D* transgenic rapeseed. Csr1-1D, a pro-197 substitution in the CSR1 catalytic subunit, confers sulfonylurea resistance by reducing the binding capacity of sulfonylurea herbicides (Haughn et al., 1988). In the present investigation, we characterized a new TBM-resistant rapeseed mutant line *K5* derived from *ZS9* by EMS mutagenesis. Molecular analysis revealed that the mutant allele *BnAHAS1^544T^* in *K5* resulted in the Pro-182-Ser replacement in BnAHAS1 (known as Pro197 in reference to *Arabidopsis*). There was no significant difference in AHAS activities of both leaves and inflorescences between *ZS9* and *K5* without TBM treatment, suggesting that the mutant allele *BnAHAS1^544T^* in *K5* did not affect the expression of this gene at the enzyme level (**Supplementary Figure S3**). Our data indicated that TBM-induced male sterility did not associate with the changes of AHAS activity of leaves, but associate with the changes of AHAS activity of inflorescences of both *ZS9* and *K5* lines, this is consistent with the point that selective male sterility is caused by differential inhibition of AHAS activity in inflorescences via transportation of the foliar-sprayed TBM previously reported by Zhao et al. (2015). The higher TBM-resistance of *K5* is target-site resistance, and this kind of resistance was caused by mutations in the target gene of the herbicide. Moreover our genetic data indicated that the TBM resistance of *K5* was controlled by a single dominant nuclear gene. Allele-specific markers developed for detecting the C-544-T variation in *BnAHAS1* did co-segregate with the herbicide resistance in rapeseed (unpublished data). Furthermore, ectopic expression of *BnAHAS1^544T^* increased TBM-tolerance of male reproductive organs of *A. thaliana.* Taken these data together, we can conclude that a higher TBM-resistance of male reproductive organs in the mutant line *K5* should be attributed to *BnAHAS1^544T^*.

Furthermore, we explored that the relationship between the male sterility (*Y*) and the changes of AHAS activity of inflorescences (*X*) can be described as a modified logistic function, *Y* = 100-*A*/(1+*Be*^(-^*^KX^*^)^) for both genotypes, although the obtained constants *A, B*, and *K* in logistic function were different for *ZS9* and *K5*. Absolute value of the *K* constant in the *ZS9* function (-0.167) is greater than that in the *K5* function (-0.125), which meaning that the percentage of sterile plants will increase more quickly for *ZS9* than *K5* with the decrease of the relative activity of AHAS of the inflorescences. We supposed that the BnAHAS activity per unit protein of the mutant line *K5* is higher than that of wild type *ZS9*, thus the catalytic efficiency of BnAHAS enzyme of the mutant line *K5* is higher than that of *ZS9* under TBM-treatment. However, this hypothesis remains to be examined in the future.

Taken together, the present study provide a novel valuable TBM-resistant rapeseed mutant line for rapeseed breeding. The higher TBM-resistance of male reproductive organs in *K5* was attributed to the Pro-182-Ser replacement in BnAHAS1. TBM-induced male sterility was associated with the relative AHAS activity of inflorescences of both genotypes. Our results supported that AHAS should be the target of AHAS-inhibiting herbicide TBM when it is used as CHA in rapeseed.

## Author Contributions

JL, SH, and HZ conceived and designed experiments. JL, QH, YS, and GQ conducted the experiments. JL and SH analyzed the data. JL, HZ, and SH wrote the manuscript. YG and XZ read the draft of the manuscript. All authors read and approved the final manuscript.

## Conflict of Interest Statement

The authors declare that the research was conducted in the absence of any commercial or financial relationships that could be construed as a potential conflict of interest.

## Footnotes

1 http://www.lynnon.com/downloads.html

2 http://www.arabidopsis.org/

## Supplementary Material

FIGURE S1 Phenotype of *ZS9* treated with different concentration of tribenuron-methly (TBM). **(A–E)** Morphological observation (upper part) and pollens viability (lower part, bars = 100 μm) of *ZS9* treated with 0, 0.05, 0.10, 1.00, and 2.00 mg⋅L^-1^ TBM. Results showed that 0.10 mg⋅L^-1^ and above TBM treatments could induce pollen abortion in *ZS9*, also inhibited growth of plants. See also **Tables 2, 3**.

FIGURE S2 Phenotype of mutant line *K5* treated with different concentration of tribenuron-methly (TBM). **(A–H)** Morphological observation (upper part) and pollens viability (lower part, bars = 100 μm) of *K5* treated with 0, 2.00, 5.00, 10.00, 15.00, 20.00, 30.00, and 40.00 mg⋅L^-1^ TBM. Results showed that 20.00 mg⋅L^-1^ and above TBM treatments could induce pollen abortion in *K5*. Also see **Table 2**.

FIGURE S3 AHAS activity of leaves and inflorescence of rapeseed *ZS9* and mutant line *K5* treated with 0 mg⋅L^-1^ tribenuron-methly. Values are the mean ± SD, *n* = 6. Different lowercase letters means significant difference at *p* = 0.05 level by Student’s *t*-test.

FIGURE S4 Phenotypes of wild type *Arabidopsis* plants 21 days after treated with different concentration of tribenuron-methly (TBM). **(A–C)** Wild type *Arabidopsis* plants treated with 0, 0.004, and 0.007 mg⋅L^-1^ TBM, respectively. **(D–F)** Pollen viability of the plants from **(A–C)**, respectively.

FIGURE S5 Phenotypes of wild type *Arabidopsis* seedlings 14 days after treated with tribenuron-methly (TBM). **(A)** 0 mg⋅L^-1^ TBM; **(B)** 0.10 mg⋅L^-1^ TBM.
