# Supplementary figures and images for: Male Sterility of an AHAS-Mutant Induced by Tribenuron-Methyl Solution Correlated With the Decrease of AHAS Activity in Brassica napus L
Source: Front Plant Sci. 2018 Jul 13;9:1014. doi: 10.3389/fpls.2018.01014 (PMC6055054; doi:10.3389/fpls.2018.01014)

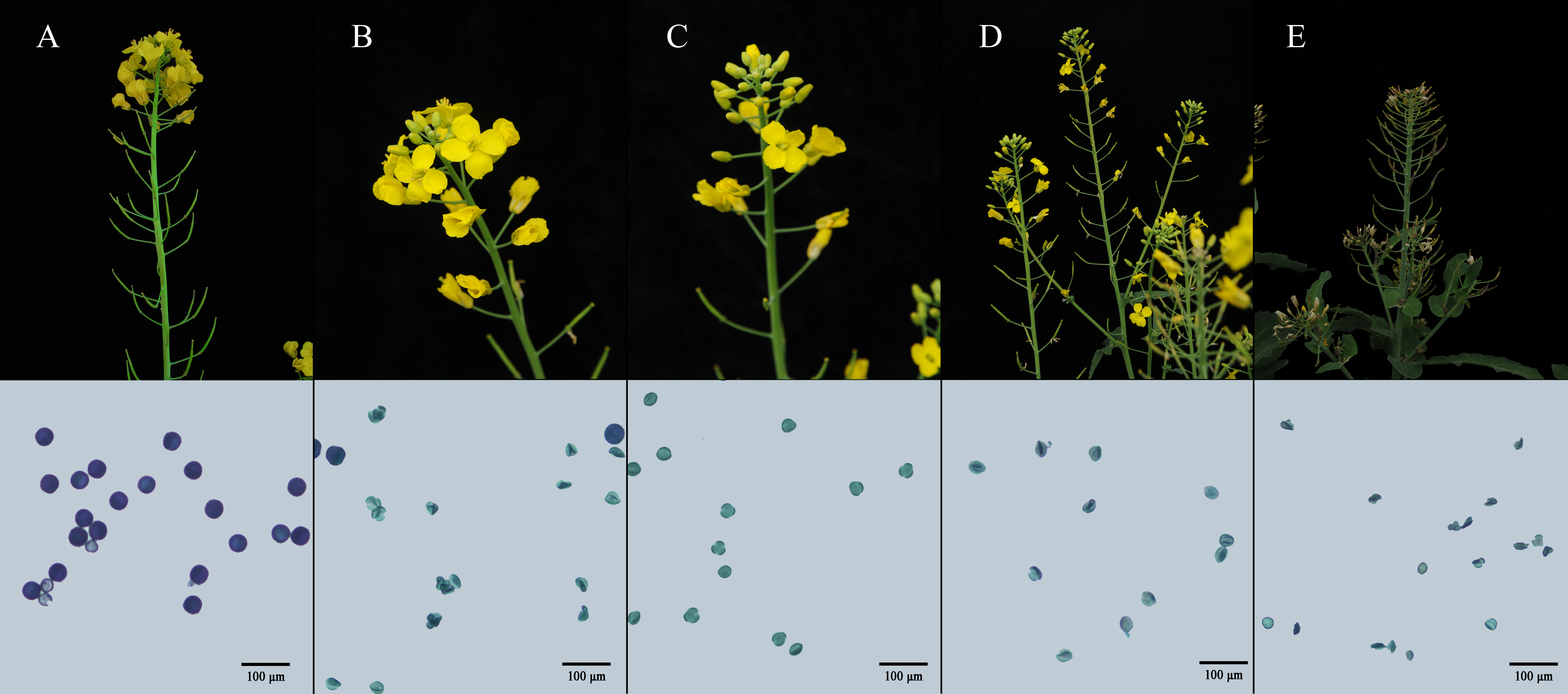

Supplement: FIGURE S1 — Phenotype of ZS9 treated with different concentration of tribenuron-methly (TBM). (A–E) Morphological observation (upper part) and pollens viability (lower part, bars = 100 μm) of ZS9 treated with 0, 0.05, 0.10, 1.00, and 2.00 mg⋅L-1 TBM. Results showed that 0.10 mg⋅L-1 and above TBM treatments could induce pollen abortion in ZS9, also inhibited growth of plants. See also Tables 2, 3. [file Image_1.JPEG]

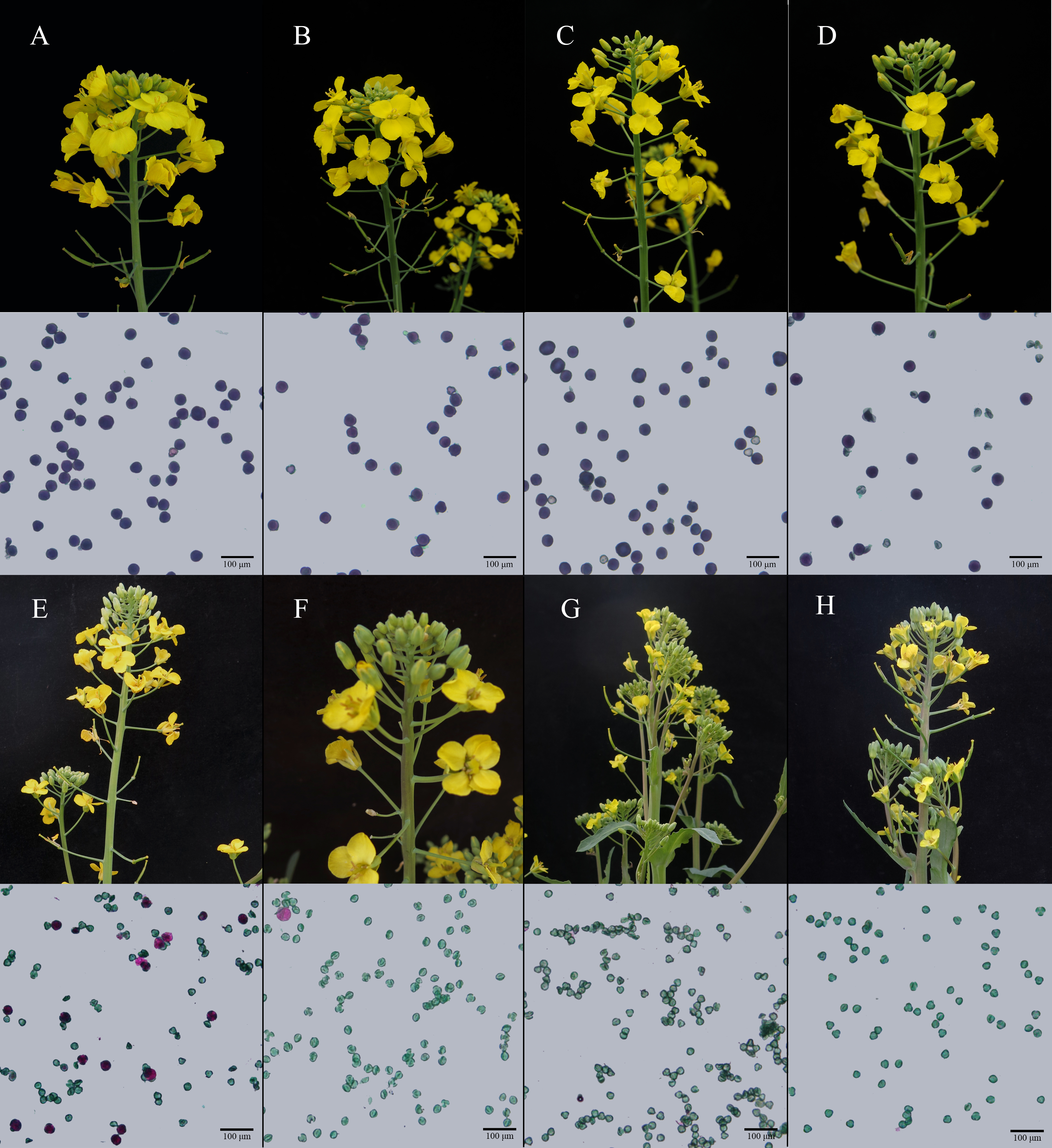

Supplement: FIGURE S2 — Phenotype of mutant line K5 treated with different concentration of tribenuron-methly (TBM). (A–H) Morphological observation (upper part) and pollens viability (lower part, bars = 100 μm) of K5 treated with 0, 2.00, 5.00, 10.00, 15.00, 20.00, 30.00, and 40.00 mg⋅L-1 TBM. Results showed that 20.00 mg⋅L-1 and above TBM treatments could induce pollen abortion in K5. Also see Table 2. [file Image_2.JPEG]

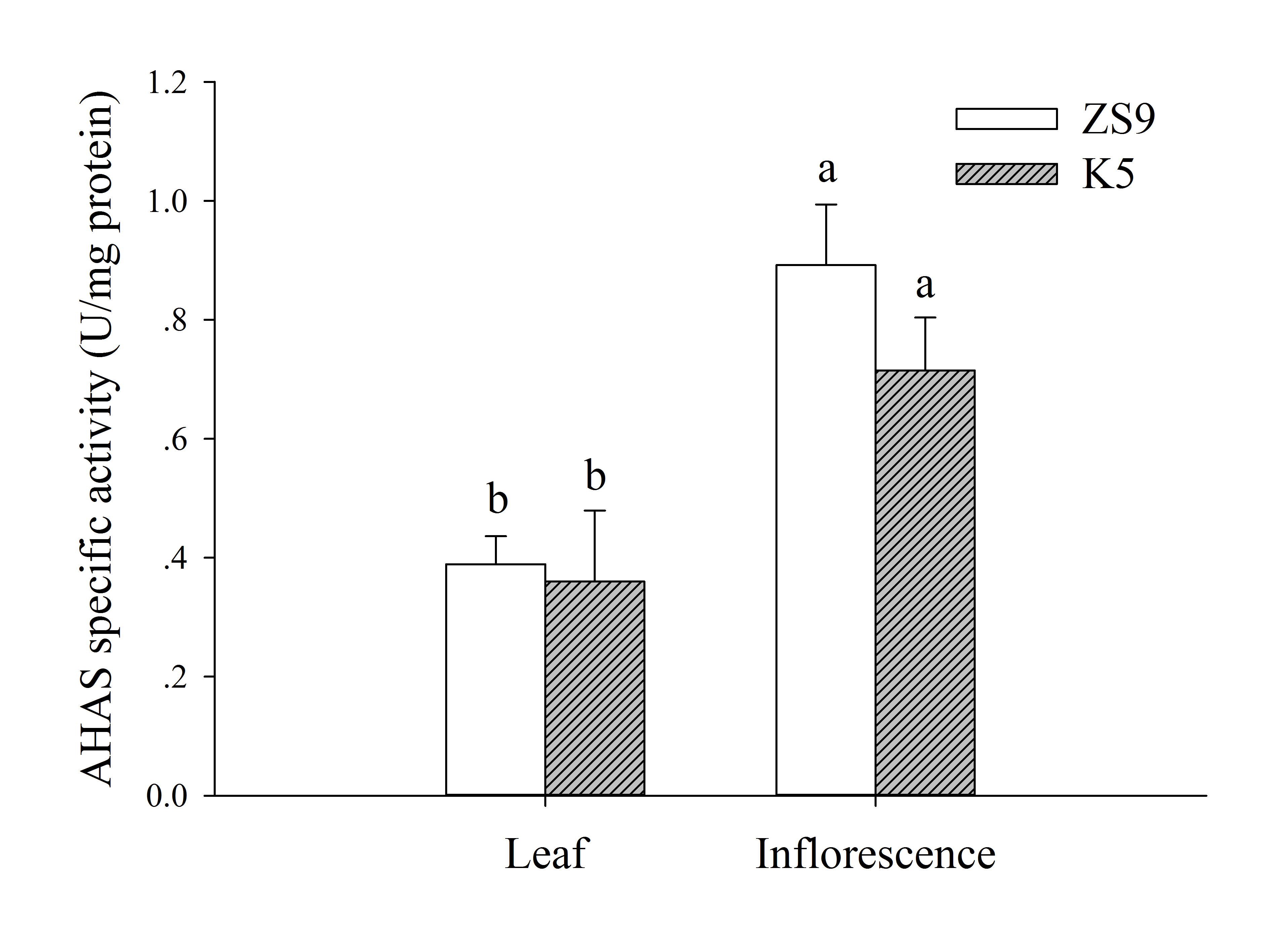

Supplement: FIGURE S3 — AHAS activity of leaves and inflorescence of rapeseed ZS9 and mutant line K5 treated with 0 mg⋅L-1 tribenuron-methly. Values are the mean ± SD, n = 6. Different lowercase letters means significant difference at p = 0.05 level by Student’s t-test. [file Image_3.JPEG]

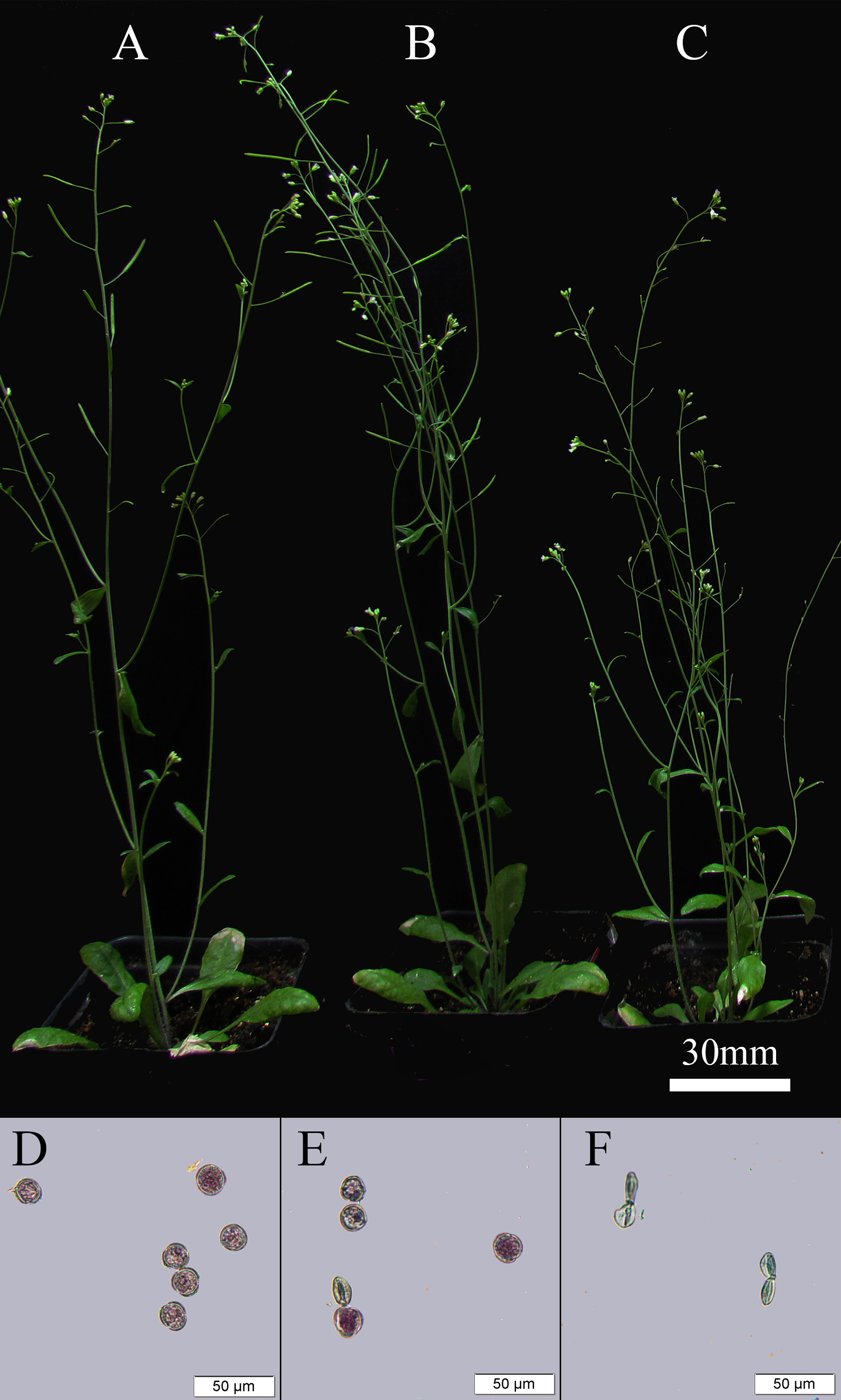

Supplement: FIGURE S4 — Phenotypes of wild type Arabidopsis plants 21 days after treated with different concentration of tribenuron-methly (TBM). (A–C) Wild type Arabidopsis plants treated with 0, 0.004, and 0.007 mg⋅L-1 TBM, respectively. (D–F) Pollen viability of the plants from (A–C), respectively. [file Image_4.JPEG]

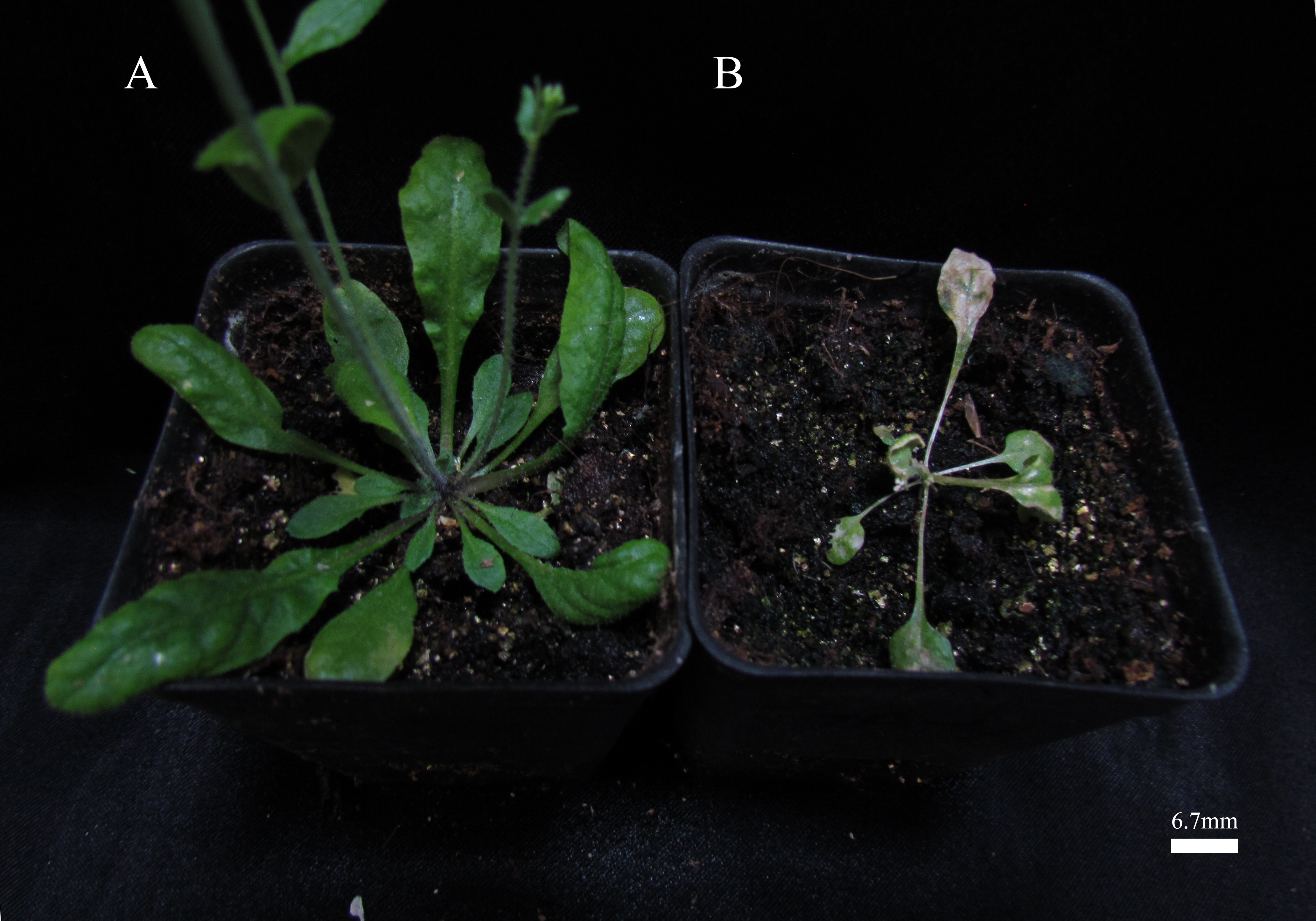

Supplement: FIGURE S5 — Phenotypes of wild type Arabidopsis seedlings 14 days after treated with tribenuron-methly (TBM). (A) 0 mg⋅L-1 TBM; (B) 0.10 mg⋅L-1 TBM. [file Image_5.JPEG]
